# Supplementary material for: Characterization of Intrinsic Radiation Sensitivity in a Diverse Panel of Normal, Cancerous and CRISPR-Modified Cell Lines
Source: Int J Mol Sci. 2023 Apr 26;24(9):7861. doi: 10.3390/ijms24097861 (PMC10178060; doi:10.3390/ijms24097861)

Characterization of intrinsic radiation sensitivity in a diverse panel of normal, cancerous and CRISPR modified cell lines

Francisco D. C. Guerra Liberal[1]; Stephen J. McMahon[1].

1- The Patrick G Johnston Centre for Cancer Research, Queen's University Belfast, Belfast, United Kingdom

Supplementary Information

Supplementary Table S1: Uncertainties values for CRISPR modified cell lines. PE – platting efficiency, SF2 – survival fraction at 2Gy, MID – mean inactivation dose, Baseline DSBs – number of foci in control samples, Induced DSBs – number of foci 30 min after 2 Gy exposure corrected for control, Residual DSBs – number of foci 24 hours after 2Gy exposure corrected for control. Standard deviation of the means for each parameter can be found in Supplementary Table S4.

**Cell line**	**Ploidy**	**Cell-cycle (%)**			**PE (%)**	**SF2**	**MID**	**Baseline DSBs**	**Induced DSBs**	**Residual DSBs**
		G1	*S*	*G2/M*						
**RPE-1**	0.05	4.00	3.08	5.45	3.58	0.05	0.09	0.15	0.71	0.42
**RPE-1 LIG4$^{-/-}$**	0.05	3.27	4.13	4.69	1.44	0.01	0.03	0.12	1.98	2.36
**RPE-1 ATM$^{-/-}$**	0.05	5.82	3.30	2.08	3.92	0.02	0.04	0.13	3.70	1.06
**RPE-1 PRKDC$^{-/-}$**	0.05	1.36	3.33	3.43	4.67	0.02	0.05	0.22	1.85	0.22
**RPE-1 DCLRE1C$^{-/-}$**	0.05	4.06	3.82	2.33	3.22	0.04	0.03	0.12	0.64	0.35
**RPE-1 TP53/BRCA1$^{-/-}$**	0.05	2.16	3.38	1.87	3.08	0.04	0.04	0.13	0.72	0.95
**RPE-1 FANCD2$^{-/-}$**	0.05	2.29	4.26	3.33	2.75	0.06	0.06	0.15	0.93	0.37
**RPE-1 BAX$^{-/-}$**	0.05	2.11	4.36	2.53	3.61	0.05	0.10	0.15	2.28	0.91
**RPE-1 TP53$^{-/-}$**	0.05	2.71	1.17	3.38	3.69	0.06	0.11	0.25	1.45	0.44

Supplementary Table S2: Uncertainties values for the cell lines used in this study. PE – platting efficiency, SF2 – survival fraction at 2Gy, MID – mean inactivation dose, Baseline DSBs – number of foci in control samples, Induced DSBs – number of foci 30 min after 2 Gy exposure corrected for control, Residual DSBs – number of foci 24 hours after 2Gy exposure corrected for control. Standard deviation of the means for each parameter can be found in Supplementary Table S5.

Cell line	*Ploidy*	*Cell-cycle (%)*			*PE (%)*	*SF2*	*MID*	*Baseline DSBs*	*Induced DSBs*	*Residual DSBs*
		G1	*S*	*G2/M*						
H441	0.42	3.01	1.10	1.96	4.37	0.06	0.06	0.35	2.46	0.56
H23	0.36	8.73	6.51	3.99	3.70	0.13	0.02	0.37	4.90	0.20
H1792	0.55	3.58	1.88	2.63	5.47	0.07	0.14	0.12	0.79	0.13
H460	0.38	3.40	2.77	4.29	2.87	0.05	0.05	0.22	4.21	0.83
SW1573	0.54	4.94	3.75	7.95	3.42	0.07	0.06	0.37	4.44	0.90
A549	0.62	5.97	2.25	6.52	4.70	0.07	0.05	0.17	0.51	0.21
C42B	0.48	3.30	3.13	3.41	1.48	0.06	0.16	0.39	2.91	0.75
PC-3	0.41	3.77	2.12	3.63	7.02	0.05	0.04	0.41	1.47	0.76
22RV1	0.35	9.47	3.26	9.95	4.79	0.07	0.04	0.21	1.96	0.07
LNCAP	0.41	4.83	1.43	5.02	1.15	0.19	0.09	0.11	1.45	0.28
DU145	0.14	4.03	1.37	2.94	7.18	0.06	0.12	0.67	0.37	0.38
MRC5	0.04	4.00	2.48	3.63	1.08	0.04	0.04	0.31	2.71	0.37
AGO1522	0.38	9.77	3.32	7.83	3.71	0.07	0.09	0.37	4.03	0.58
RPE-1	0.05	4.00	3.08	5.45	3.58	0.05	0.09	0.15	0.71	0.42
MCF10A	0.28	9.41	1.72	9.39	1.76	0.06	0.07	0.15	1.01	0.65
PNT1A	0.33	1.61	2.04	0.96	6.34	0.07	0.17	0.41	2.09	0.37
RWPE1	0.08	5.96	2.82	3.64	1.75	0.07	0.08	0.40	4.25	0.99
WPMY-1	0.25	6.11	2.71	3.80	3.26	0.05	0.12	0.29	3.63	0.76
PNT2	0.52	2.18	1.06	2.33	2.26	0.06	0.06	0.09	0.74	0.86

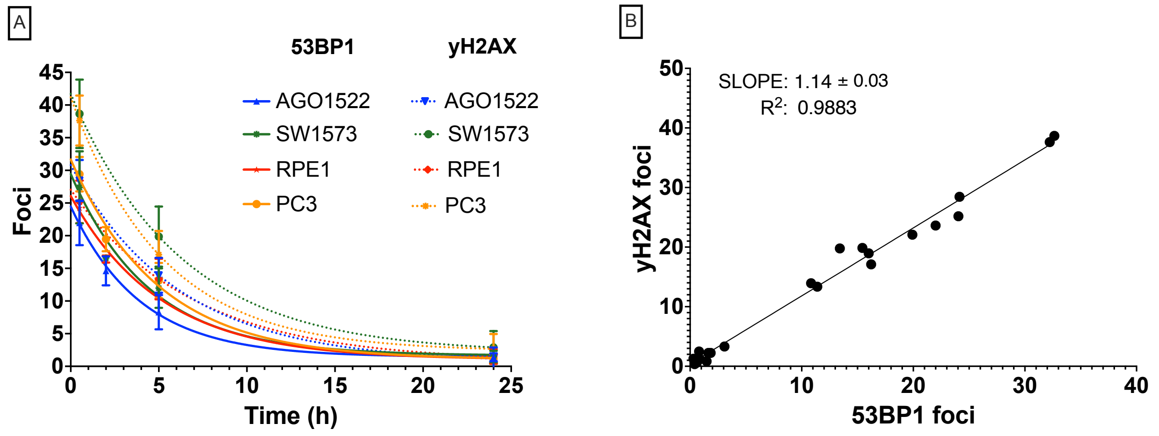

Supplementary Figure S1: (A) Repair kinetics of radiation-induced foci measured by two different DSBs markers 53BP1 and yH2AX in two cell lines with highest ploidy value and two cell lines with ploidy value of 2. (B) Correlation of the values measured by each DSBs marker.

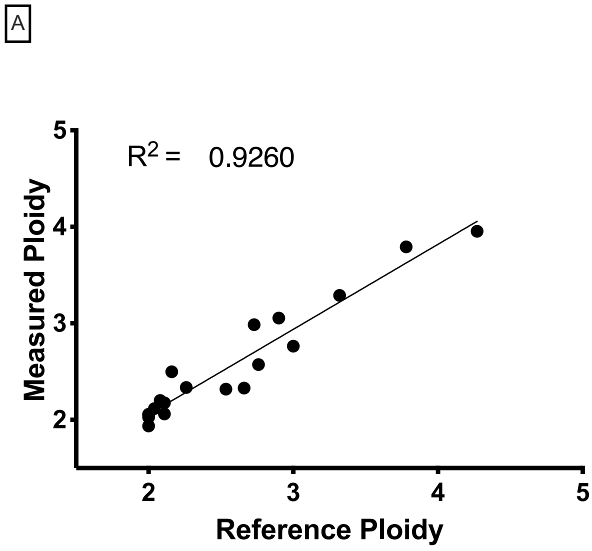

B

	Reference	Measured
A549	2.7600	2.572386111
LNCAP	3.7800	3.791796602
22RV1	2.0400	2.115682599
AGO1522	2.0000	2.027210105
C42B	3.0000	2.763081764
DU145	2.6600	2.328469967
H1792	2.1600	2.497178678
H23	2.9000	3.053928253
H460	2.5350	2.317223711
MCF10A	2.0000	2.052562774
MRC5	2.0000	2.057350473
PC3	4.2700	3.953592509
PNT2	2.1100	2.176076265
RPE1	2.0000	1.936222941
RWPE1	2.0800	2.200338631
SW1573	3.3200	3.289024797
WMPY1	2.7300	2.985348575
H441	2.2608	2.334430000
PNT1A	2.1100	2.060788400

Supplementary Figure S2: (A) Correlation between ploidy values measured in this study in ploidy values reported by the distributed; (B) Values used to perform the correlation in the subplot A.

Supplementary Table S3: Coefficient of determination and p-values associated with the correlation between different metrics and MID used to produce Figure 7.

Cell line	*Complete Set*		*Lung*		*Prostate*		*Normal*	
	R^2	p-value	R^2	p-value	R^2	p-value	R^2	p-value
Plating Efficiency	0.313	0.013	0.789	0.018	0.033	0.770	0.228	0.231
Ploidy	0.079	0.244	0.147	0.452	0.020	0.821	0.359	0.117
G1 (%)	0.090	0.212	0.416	0.167	0.019	0.818	0.451	0.068
S (%)	0.374	0.005	0.551	0.091	0.087	0.632	0.414	0.085
G2 (%)	0.016	0.605	0.724	0.032	0.005	0.907	0.352	0.121
Baseline DSB	0.001	0.992	0.640	0.056	0.002	0.935	0.163	0.321
Induced DSB	0.012	0.648	0.009	0.860	0.696	0.078	0.067	0.534
Residual DSB	0.022	0.542	0.220	0.348	0.156	0.510	0.017	0.760

Organ	Prostate	Prostate	Prostate	Prostate	Prostate	Lung	Lung	Lung	Lung	Lung	Lung	Lung
Condition	Carcinoma	Carcinoma	Carcinoma	Carcinoma	Adenocarcinoma	Carcinoma	Carcinoma	Carcenoma	Adenocarcinoma	Adenocarcinoma	Adenocarcinoma	Adenocarcinoma
Metastatic site	-	Brain	LnCap co-inoculated	lymph nod	Bone	-	Pleural Effusion	Lymph Node	-	-	-	Pleural Effusion
Cell Line	22Rv1	Du145	C4-2B	LNCaP	PC-3	A549	H460	H1299	H23	H441	SW1573	H1792
Morphology	epithelial	epithelial	epithelial	epithelial	epithelial	epithelial	epithelial	epithelial	epithelial	epithelial	epithelial	
ATM	Missense Mutation	WT	WT	Nonsense_Mutation	WT	WT	WT	WT	Missense Mutation	WT	WT	WT
ATR	Frame Shift Ins	WT	WT	Silent	WT	Splice Site	WT	WT	WT	WT	WT	WT
BAX (BCL2)	Frame Shift Del	Frame Shift Ins	WT	Frame Shift Del	WT	WT	WT	WT	WT	WT	WT	WT
BRCA1	WT	Missense Mutation	WT	WT	WT	WT	WT	WT	WT	WT	WT	WT
BRCA2	Missense Mutation	Missense Mutation	WT	WT	WT	WT	WT	WT	WT	Silent	WT	WT
CDK12	WT	WT	WT	WT	WT	WT	WT	WT	WT	WT	WT	WT
CHEK1	WT	WT	WT	WT	WT	WT	WT	WT	WT	WT	WT	WT
CHEK2	WT	WT	WT	Missense Mutation	WT	WT	WT	WT	WT	WT	WT	WT
DCLRE1C (Artemis)	Frame Shift Del	Silent	WT	WT	WT	WT	WT	WT	Silent	WT	WT	WT
FACG	WT	WT	WT	WT	WT	WT	WT	WT	WT	WT	WT	WT
FANC(A/B/C/D2/E/F/G/	(M) Silent	(B/I) Missense Mutation	WT	(A/B) Missense Mutati	WT	WT	WT	WT	(I) Missense Mutation	(M) Silent	WT	WT
FLIP (CFLAR)	WT	WT	WT	WT	WT	WT	WT	WT	WT	WT	WT	WT
H2AFX	WT	WT	WT	WT	WT	WT	WT	WT	WT	WT	WT	WT
LIG4	Silent	Missense Mutation	WT	WT	WT	WT	WT	WT	WT	WT	WT	WT
MRE11A	WT	WT	WT	WT	WT	WT	WT	WT	WT	Missense Mutation	WT	WT
NBN (NBS1)	Missense Mutation	WT	WT	WT	WT	WT	Missense Mutation	WT	Missense Mutation	WT	WT	WT
NHEJ1 (XLF)	WT	WT	WT	Missense Mutation	WT	WT	WT	WT	WT	WT	WT	WT
PALB2 (FANCN)	Missense Mutation	WT	WT	WT	WT	WT	WT	WT	WT	Nonsense_Mutation	WT	WT
PARP1	WT	WT	WT	WT	WT	WT	WT	WT	WT	WT	WT	WT
PRKDC (XRCC7) (DNA-PK	WT	Frame Shift Ins	WT	Silent	WT	WT	WT	WT	WT	Missense Mutation	WT	WT
PTEN	WT	Missense Mutation	Silent	Frame Shift Del	Silent	WT	WT	WT	WT	WT	WT	WT
RAD50	Missense Mutation	Frame Shift Del	WT	Frame Shift Del	WT	WT	WT	WT	WT	WT	WT	WT
RAD51	WT	WT	WT	WT	WT	WT	WT	WT	WT	WT	WT	WT
RBBP8 (CTIP)	WT	Missense Mutation	WT	Frame Shift Ins	WT	WT	WT	WT	WT	WT	WT	WT
TP53	Frame Shift Del	Missense Mutation	Missense Mutation	Silent	Frame Shift Del	WT	WT	Silent	Missense Mutation	Missense Mutation	WT	Splice Site
TP53BP1	Frame Shift Del	Splice Site	WT	Nonsense_Mutation	WT	Splice Site	WT	WT	WT	WT	WT	WT
XRCCU5 (KU80)	WT	WT	WT	WT	WT	WT	WT	WT	WT	WT	WT	WT
XRCCU6 (KU70)	WT	WT	WT	WT	WT	WT	WT	WT	WT	WT	WT	WT

Supplementary Table S4: List of mutations in key genes of DNA damage response system in the cell lines used in this study.

Table S5: Cell lines origin and culture media.

Cell line	*Origin*	*Age (y)*	*Ploidy*	*Karyotype*	*Morphology*	*Doubling time (h)*	*Medium*	*Supplements*
H460	Lung - Male	-	2.5	57	Epithelial	18	RPMI-1640	10% FBS; 1% Pen-Strep
A549	Lung - Male	58	2.8	66	Epithelial	23	RPMI-1640	10% FBS; 1% Pen-Strep
H1792	Lung - Male	50	2.2	-	Epithelial	32	RPMI-1640	10% FBS; 1% Pen-Strep
SW1573	Lung - Female	44	3.3	-	Epithelial	23	RPMI-1640	10% FBS; 1% Pen-Strep
H23	Lung - Male	51	2.9	-	Epithelial	34	RPMI-1640	10% FBS; 1% Pen-Strep
H441	Lung - Male	33	2.3	52	Epithelial	58	RPMI-1640	10% FBS; 1% Pen-Strep
PC-3	Prostate - Male	62	4.3	62	Epithelial	27	RPMI-1640	10% FBS; 1% Pen-Strep
DU145	Prostate - Male	69	2.7	61	Epithelial	32	RPMI-1640	10% FBS; 1% Pen-Strep
22RV1	Prostate - Male	-	2.0	-	Epithelial	49	RPMI-1640	10% FBS; 1% Pen-Strep
C42B	Prostate - Male	50	3.0	-	Epithelial	36	RPMI-1640	10% FBS; 1% Pen-Strep
LNCAP	Prostate -Male	50	3.8	84	Epithelial	59	RPMI-1640	10% FBS; 1% Pen-Strep
PNT1A	Normal Prostate	35	2.1	-	Epithelial immortalized with SV40	30	RPMI-1640	10% FBS; 1% Pen-Strep
MRC5	Normal Lung	14 w	2.0	46	Fibroblast	36	RPMI-1640	10% FBS; 1% Pen-Strep
PNT2	Normal Prostate	33	2.1	-	Epithelial immortalized with SV40	36	RPMI-1640	10% FBS; 1% Pen-Strep
WPMY1	Normal Prostate	54	2.7	62	Myofibroblast immortalized with SV40	22	DMEM	5% FBS; 1% Pen-Strep
AGO1522	Normal Skin	3 d	2.0	46	Fibroblast	25	EMEM	15% FBS; 1% Pen-Strep
RPE-1	Normal Retina	1	2.0	46	Epithelial immortalized with hTERT	18	DMEM:F12	10% FBS; 1% Pen-Strep
MCF10A	Normal Breast	36	2.0	-	Epithelial	27	DMEM:F12	5% Horse Serum, 20 ng/ml Hydrocortisone, 100 ng/ml Cholera Toxin, 10 ug/ml Insulin, 1% Pen-Strep
RWPE1	Normal Prostate	54	2.1	46	Epithelial immortalized with HPV-18	22	Keratinocyte Serum Free Medium	0.05 mg/ml of bovine pituitary extract, 5 ng/ml epidermal growth factor

Table S6: Guide crRNA for CRISPR/Cas9.

Gene	*Sequence*	*IDT identification nº*
TP53	CCATTGTTCAATATCGTCCG	Hs.Cas9.TP53.1.AA
ATM	ATTTATATCCATCATCCGAA	Hs.Cas9.ATM.1.AA
LIG4	GCTTATACGGATGATCATAA	Hs.Cas9.LIG4.1.AA
BRCA1	GACGTCTGTCTACATTGAAT	Hs.Cas9.BRCA1.1.AA
FANCD2	AGTTGACTGACAATGAGTCG	Hs.Cas9.FANCD2.1.AA
BAX	ACTCGGAAAAAGACCTCTCG	Hs.Cas9.BAX.1.AA
PRKDC (DNA-PKcs)	GAACTTCACCCAATAATCCT	Hs.Cas9.PRKDC.1.AB
DCLRE1C (Artemis)	TGGTCCGAAGCTGGATCACT	Hs.Cas9.DCLRE1C.1.AB

Supplementary Figure S3: DDR proteins Knock-out validation by Western Blot (A) BAX, (B) Artemis, (C) BRCA1, (D) DNA-LIG4, (E) ATM, (F) p53 and (H) FANCD2.

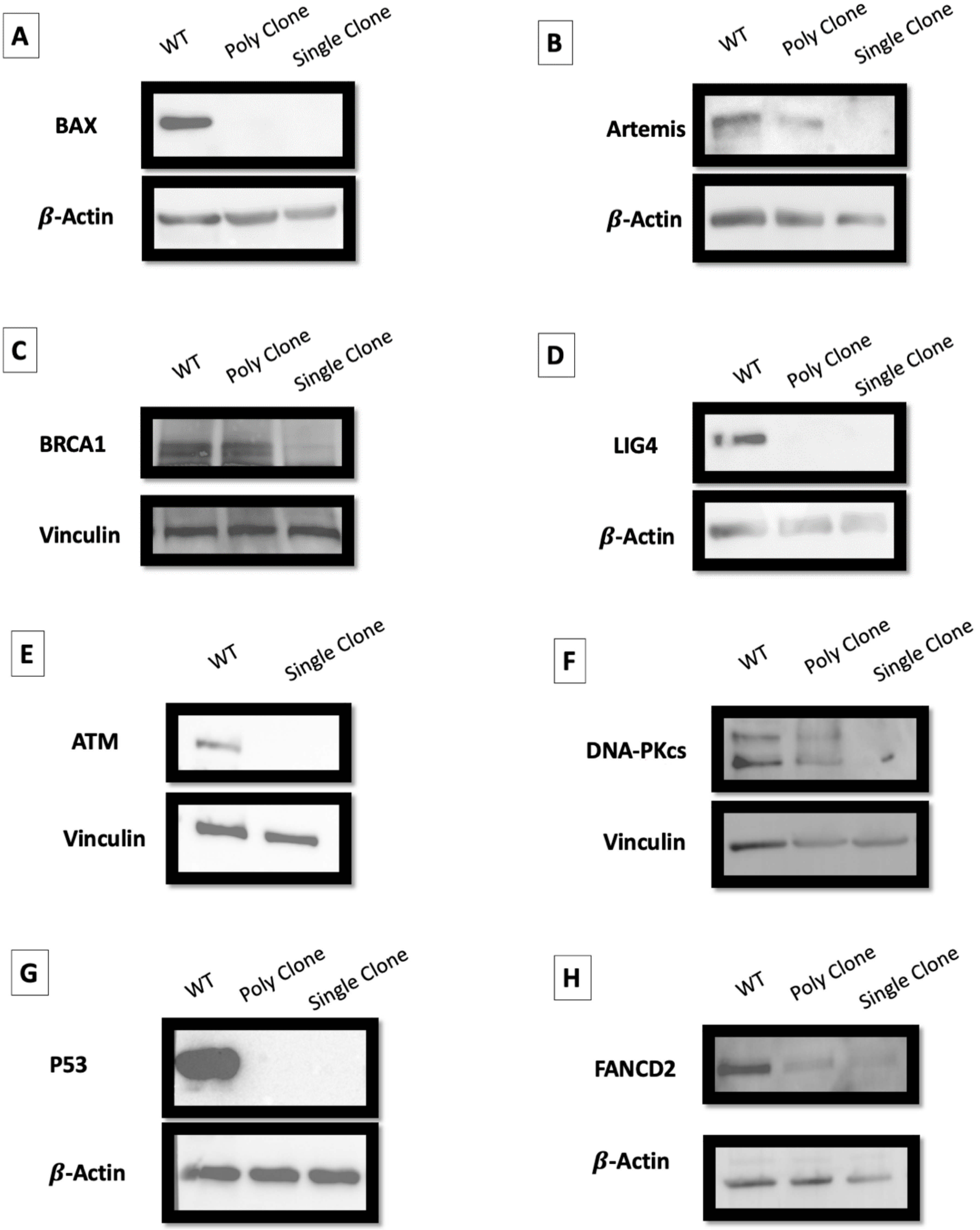

Table S7: List of Antibodies.

Antigen	*Source*	*Type*	*Dilution*
P53	Santa Cruz	(DO-1) monoclonal	1:500
ATM	Santa Cruz	(G-12) monoclonal	1:500
Artemis	Cell Signalling	(D5N5N) monoclonal	1:1000
BAX	Cell Signalling	(2772) polyclonal	1:1000
BRCA1	Santa Cruz	(D9) monoclonal	1:100
FANCD2	Santa Cruz	(FI17) monoclonal	1:500
DNA-PKcs	Cell Signalling	(3H6) monoclonal	1:500
53BP1	Novus Biologicals	(304) polyclonal	1:5000
BAX	Millipore	(JBW301) monoclonal	1:5000

Supplementary Figure S4: Representative images of 53BP1 immunofluorescence in RPE-1 wild-type, RPE-1 p53 $^{-/-}$, RPE-1 LIG4 $^{-/-}$ and RPE-1 p53 $^{-/-}$/ RPE-1 BRCA1 $^{-/-}$.

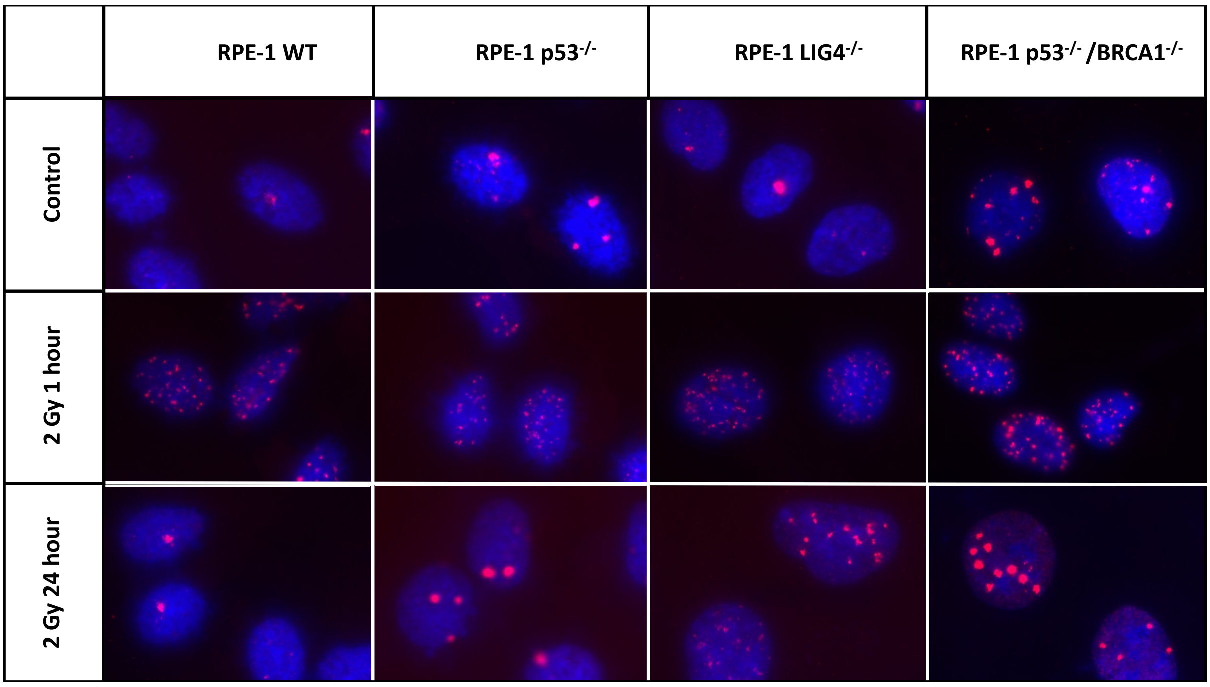

Supplement: Supplementary file 1 [file ijms-24-07861-s001.zip › SI/Characterization SI .pdf]
